# Supplementary figures and images for: The effect of compound kushen injection on cancer cells: Integrated identification of candidate molecular mechanisms
Source: PLoS One. 2020 Jul 30;15(7):e0236395. doi: 10.1371/journal.pone.0236395 (PMC7392229; doi:10.1371/journal.pone.0236395)

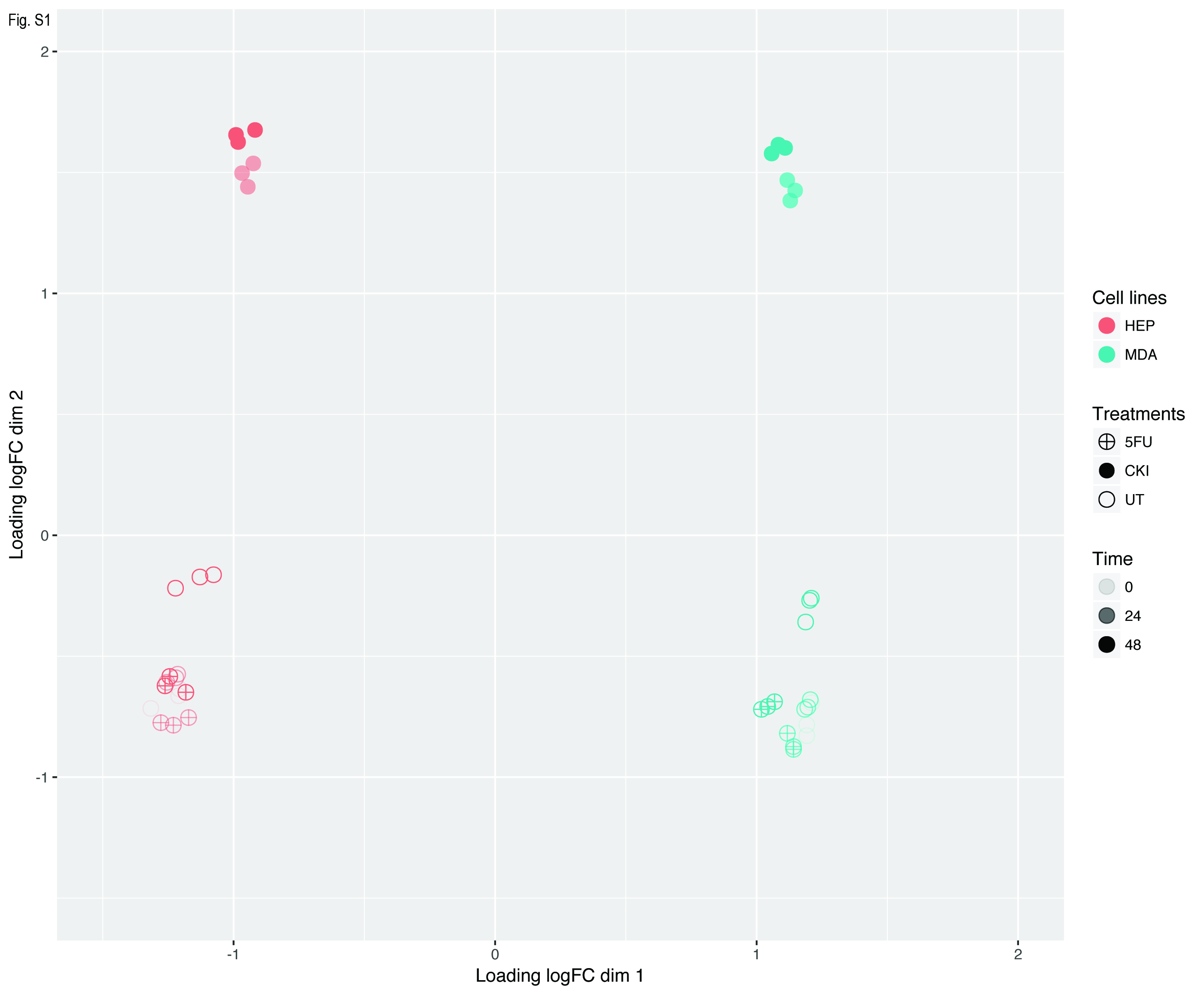

Supplement: S1 Fig — (TIF) [file pone.0236395.s001.tif]

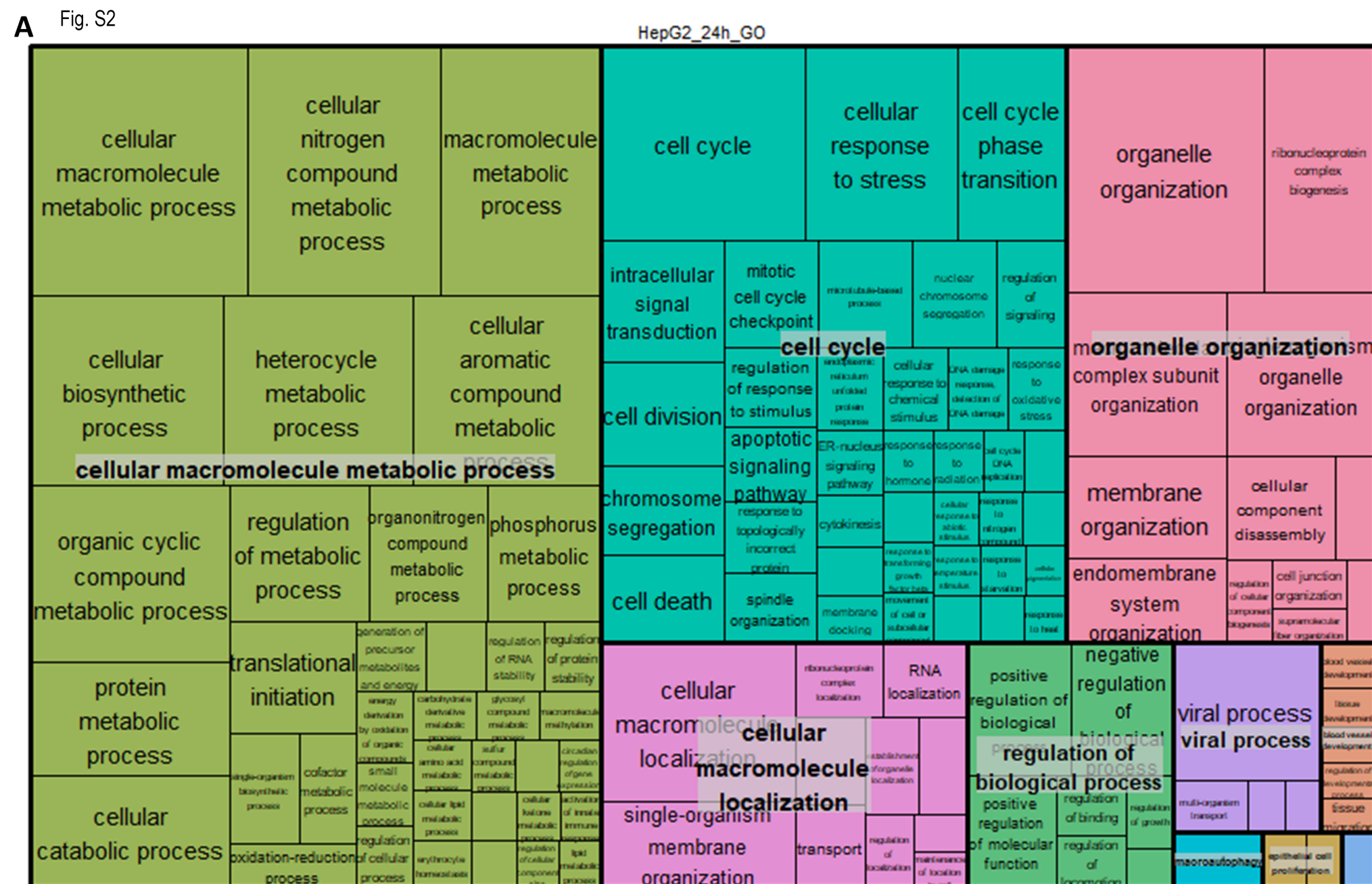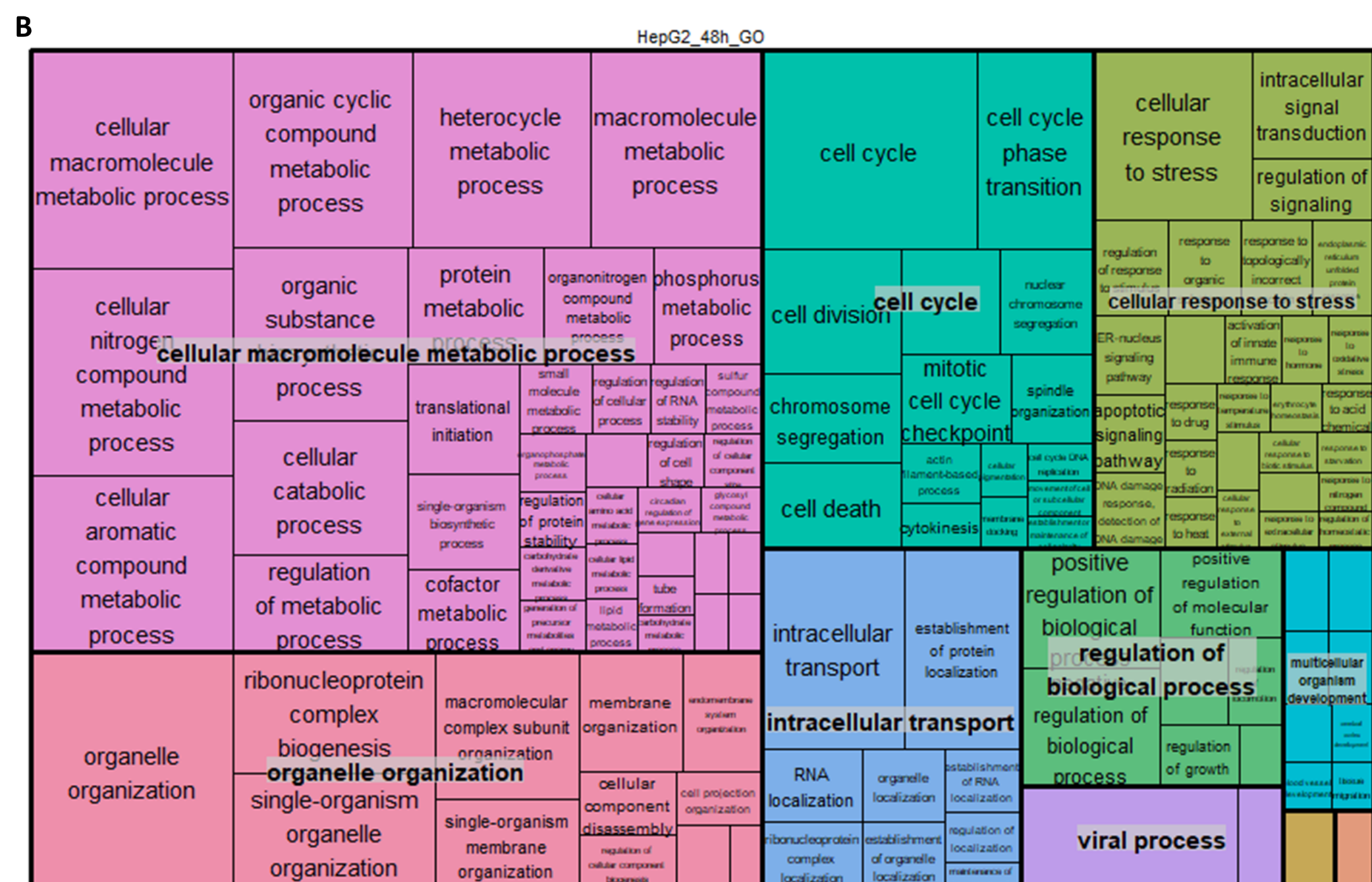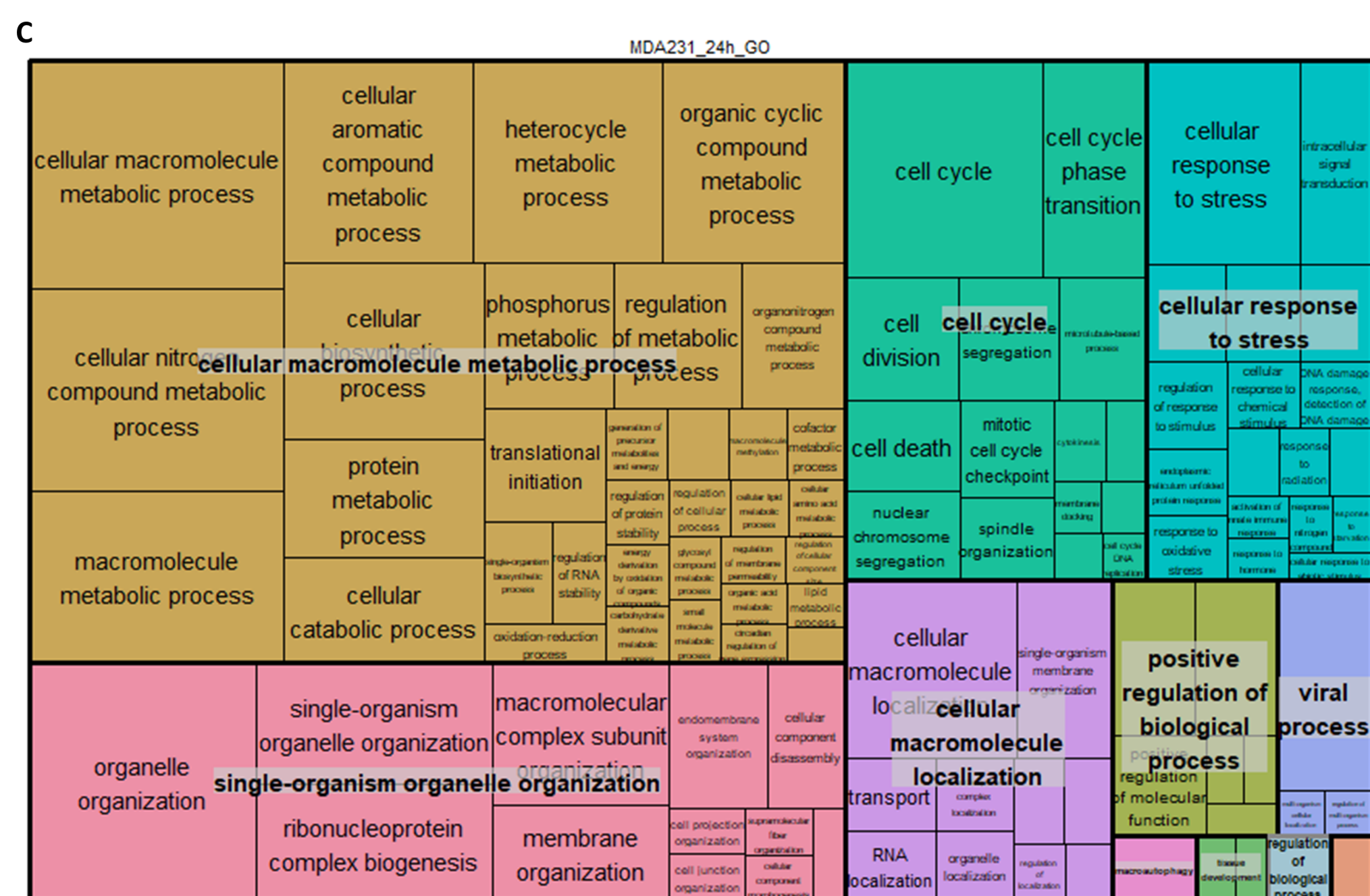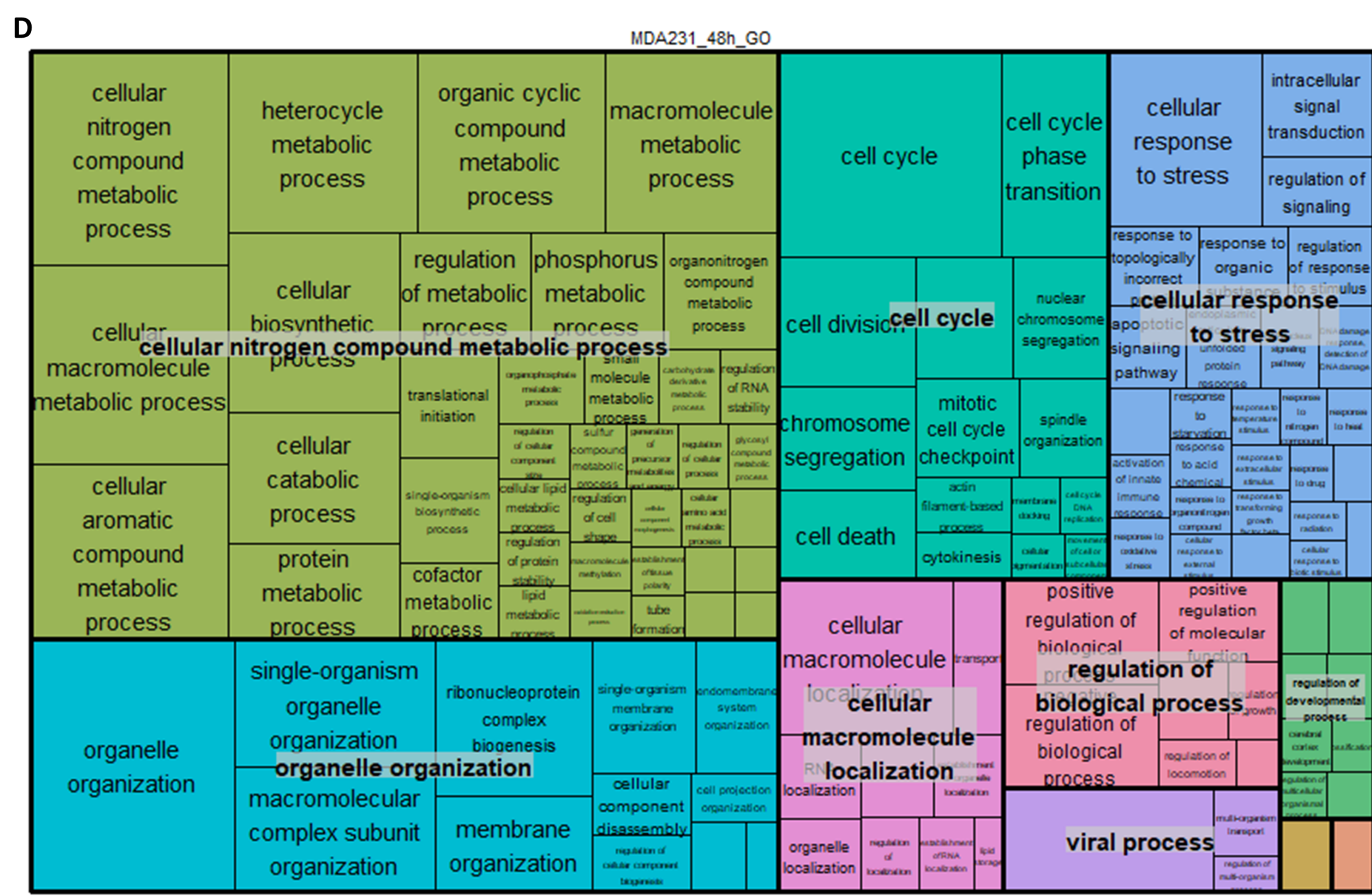

Supplement: S2 Fig — (A-D) Each small square represents a GO biological process function at level 3. Size of the squares positively correlates with the statistical significance of related biological process. Different colours distinguish biological process clusters that are described by the top shaded functional representatives. (PDF) [file pone.0236395.s002.pdf]

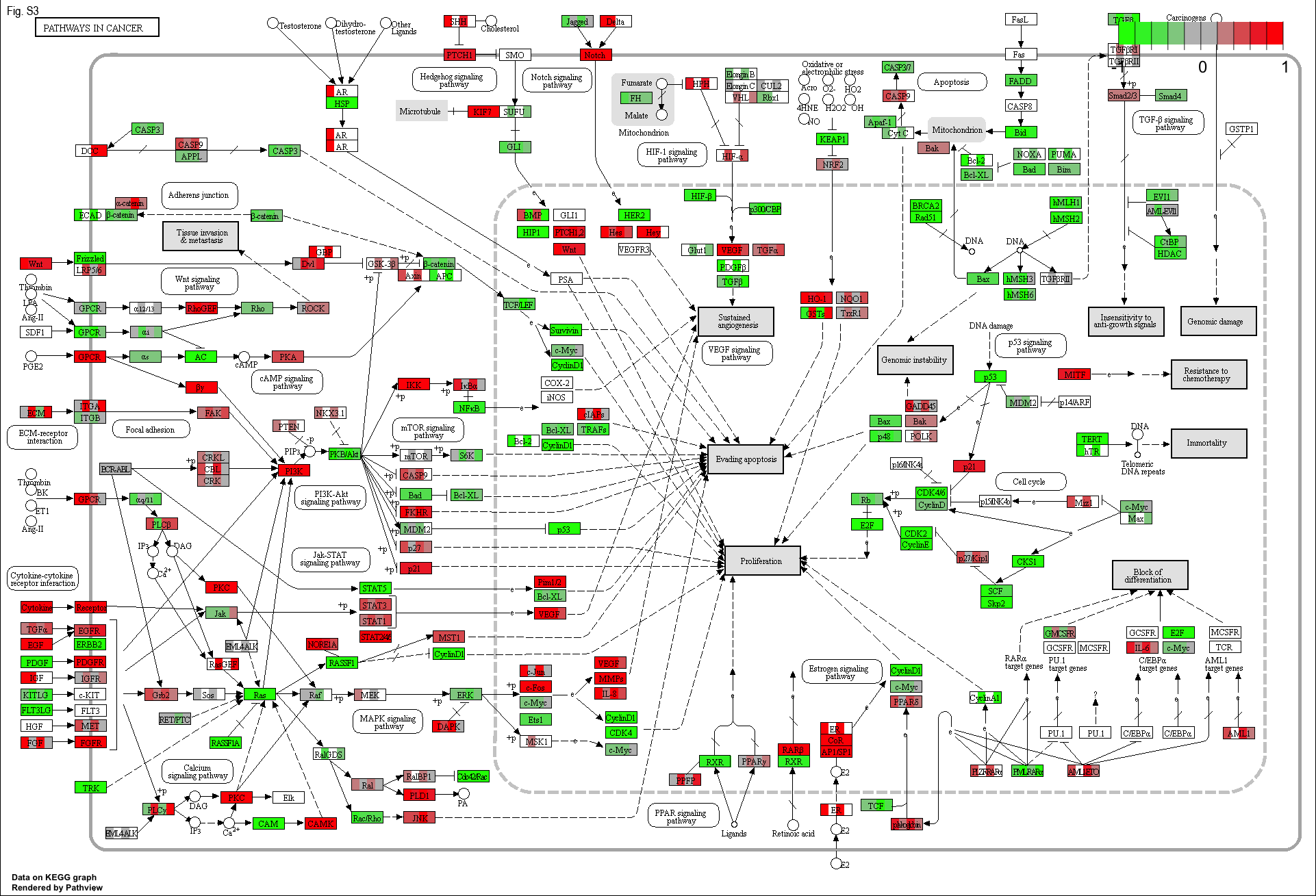

Supplement: S3 Fig — In the cell cycle pathway, each coloured box is separated into 4 parts, from left to right representing 24h CKI treated Hep G2, 48h CKI treated Hep G2, 24h CKI treated MDA-MB-231 and 48h CKI treated MDA-MB-231. (TIF) [file pone.0236395.s003.tif]

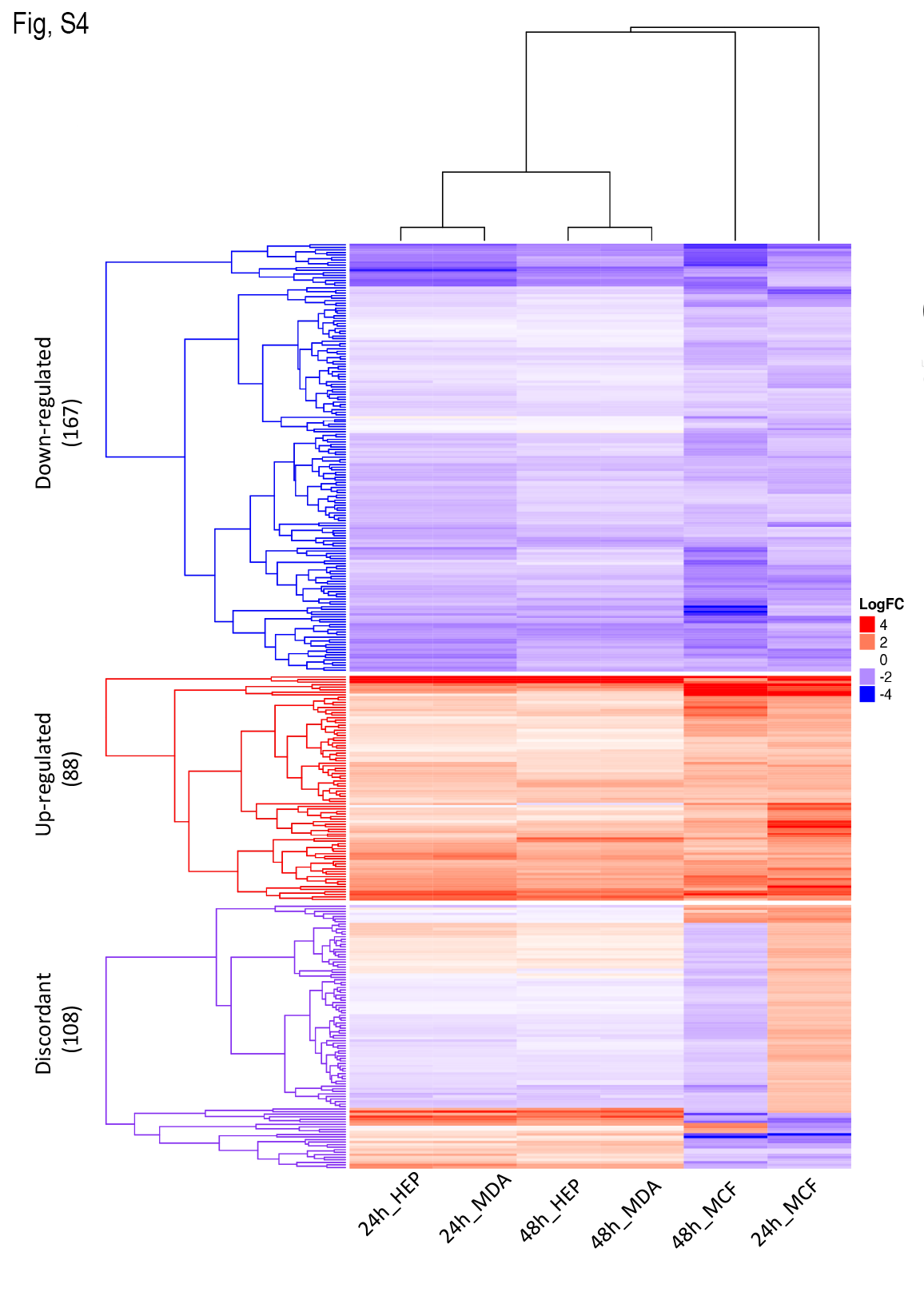

Supplement: S4 Fig — Heatmap showing the expression fold changes of core genes in three cell lines at two time points. All the core genes can be separated into the following 3 clusters: genes up regulated in all three cell lines, genes down regulated in all three cell lines and DE genes that are uncorrelated in terms of expression change across the three cell lines. (TIF) [file pone.0236395.s004.tif]
