# Supplementary material for: The effect of compound kushen injection on cancer cells: Integrated identification of candidate molecular mechanisms
Source: PLoS One. 2020 Jul 30;15(7):e0236395. doi: 10.1371/journal.pone.0236395 (PMC7392229; doi:10.1371/journal.pone.0236395)
Supplement: S1 File — Both original Chinese document and English translation included. (PDF) [file pone.0236395.s009.pdf]

Quality Control (QC) report for CKI (Batch No. 20150404)

**Product Name**	Compound Kushen Injection (CKI)	**Volume**	5 ml	**Formulation**	Injection
**Manufacture workshop Number**	1	**Batch Number**	20150404	**Total Quantity**	13,074
**Sampling date**	04/04/2015	**Code**	CJ150404	**Report Number**	C150404
**QC standards**	ZGB2014-16			**Report Date**	24/05/2015

**QC items**	**Standard**	**Result**
**Traits**	Yellow-brown or red-brown orange-red liquid	Yellow-brown or red-brown orange-red liquid
**Identification**		
1 Thin Layer Chromatography	In the chromatogram of the test sample, the same three orange-red spots should be displayed at the positions corresponding to the chromatograms of matrine, sophoridine and oxymatrine.	Meet the criteria
2 Liquid Chromatography	At the retention time of the macrozamin reference solution, the test solution should have a consistent peak.	Meet the criteria
**Inspection**		
pH	7.0~7.8	7.4
Color	should meet standard*	Yes
Burning residue	<= 4.5% (g/ml)	3.1%
Total solids	>= 8.5% (g/ml)	14.4%
Relative substance	should meet standard*	Yes
Quantity	should meet standard*	Yes
Visible particles	should not be detected	not detected
Insoluble particles	should meet standard*	Yes
Polysorbate 80	<= 2.6mg/ml	2.4mg/ml
Abnormal toxicity	should meet standard*	Yes
Pyrogen	should meet standard*	Yes
Depressor substance	should meet standard*	Yes
Allergic reaction	should meet standard*	Yes
Hemolysis and cohesion	should meet standard*	Yes
Heavy metals and toxic substances	should meet standard*	Yes
Fingerprint	According to the similarity evaluation system of traditional Chinese medicine chromatographic fingerprint,	0.97

	the similarity value between the fingerprint of the test sample and the reference should be >= 0.92	
Content determination		
Kushen	Total content of matrine ($C_{15}H_{24}N_2O$) and oxymatrine ($C_{15}H_{24}N_2O_2$) per 1ml CKI should be 9.0mg ~14.0 mg	12.4mg
Baituling	Content of macrozamin ($C_{13}H_{24}N_2O_{11}$) perl 1ml CKI, should be 0.70mg~1.10mg	0.78 mg

QC result	Pass	Remarks	N/A

Signature: Ying Li Date: 24/052015

Second batch 19/11/2015

振东制药
ZHENDONG PHARMACEUTICAL

文件编号：Z-SFP01153(00)

成品检验报告单 Quality control report

品　名	复方苦参注射液	剂　型	注射剂	包装规格	5ml×5 支×100 盒
批　号	20150404	代　码	CP0001	箱　数	129 件
生产车间	小容量注射剂 1 车间	检验目的	出厂检验	数　量	13074 盒
取样日期	2015 年 04 月 04 日	检验单号	CJ150404	报告单号	C150404
检验依据	国家食品药品监督管理总局国家药品标准（修订）颁布件 ZGB2014-16			报告日期	2015 年 05 月 24 日

检验项目及结果

检验项目	标准规定	检验结果
【性　状】	本品应为黄棕色至红棕色的澄明液体	本品为黄棕色至红棕色的澄明液体
【鉴　别】		
(1) 薄层色谱	供试品色谱中，在与苦参碱、槐定碱及氧化苦参碱对照品色谱相应的位置上，应显相同的三个橘红色斑点。	供试品色谱中，在与苦参碱、槐定碱及氧化苦参碱对照品色谱相应的位置上，显相同的三个橘红色斑点。
(2) 液相色谱	在甲基氧化偶氮甲醇樱草糖苷对照品溶液相应的保留时间处，供试品溶液应有保留时间一致的色谱峰。	在甲基氧化偶氮甲醇樱草糖苷对照品溶液相应的保留时间处，供试品溶液有保留时间一致的色谱峰。
【检　查】		
pH　值	应为 6.8～7.8	7.4 ph=7.4
溶液颜色	应符合规定	符合规定
炽灼残渣	应≤5.0%（g/ml）	3.1%
总固体	应≥8.0%（g/ml）	14.4%
有关物质	应符合规定	符合规定
装　量	应符合规定	符合规定
可见异物	应不得检出	未检出
无　菌	应符合规定	符合规定
不溶性微粒	应符合规定	符合规定
聚山梨酯 80	本品每 1ml 含聚山梨酯 80 不得过 2.7mg	2.4mg
异常毒性	应符合规定	符合规定
热　原	应符合规定	符合规定
降压物质	应符合规定	符合规定
过敏反应	应符合规定	符合规定
溶血与凝聚	应符合规定	符合规定
重金属及有害元素残留量	应符合规定	符合规定
【指纹图谱】	按中药色谱指纹图谱相似度评价系统，供试品指纹图谱与对照指纹图谱经相似度计算，相似度不得低于 0.90	0.97
【含量测定】		
苦　参 (Kushen)	每 1ml 含苦参以苦参碱（$C_{15}H_{24}N_2O$）和氧化苦参碱（$C_{15}H_{24}N_2O_2$）的总量计，应为 8.0mg～14.0mg	12.4mg (12.4mg/ml Matrine + Oxymatrine)
白土苓 (Baituling)	每 1ml 含白土苓以甲基氧化偶氮甲醇樱草糖苷（$C_{13}H_{24}N_2O_{11}$）计，应为 0.35mg～1.20mg	0.78mg (Macrozamin 0.78mg/ml)

结果判定	本品按国家食品药品监督管理总局国家药品标准（修订）颁布件 ZGB2014-16 检验，结果符合规定。	备注	

BS3347-11

负责人/日期：李英 2015年05月24日　复核人/日期：张彦淑 2015年05月24日　检验人/日期：[illegible] 范佳丽 [illegible] 2015年05月24日
